# Pro-Inflammatory wnt5a and Anti-Inflammatory sFRP5 Are Differentially Regulated by Nutritional Factors in Obese Human Subjects

**DOI:** 10.1371/journal.pone.0032437

**Published:** 2012-02-23

**Authors:** Dominik M. Schulte, Nike Müller, Katrin Neumann, Frank Oberhäuser, Michael Faust, Heike Güdelhöfer, Burkhard Brandt, Wilhelm Krone, Matthias Laudes

**Affiliations:** 1 Department I of Internal Medicine, University of Kiel, Kiel, Germany; 2 Center of Endocrinology, Diabetes and Preventive Medicine, University of Cologne (Germany), Köln, Germany; 3 Institute for Clinical Chemistry, University of Kiel, Kiel, Germany; University of Cambridge, United Kingdom

## Abstract

**Background:**

Obesity is associated with macrophage infiltration of adipose tissue. These inflammatory cells affect adipocytes not only by classical cytokines but also by the secreted glycopeptide wnt5a. Healthy adipocytes are able to release the wnt5a inhibitor sFRP5. This protective effect, however, was found to be diminished in obesity. The aim of the present study was to examine (1) whether obese human subjects exhibit increased serum concentrations of wnt5a and (2) whether wnt5a and/or sFRP5 serum concentrations in obese subjects can be influenced by caloric restriction.

**Methodology:**

23 obese human subjects (BMI 44.1±1.1 kg/m^2^) and 12 age- and sex-matched lean controls (BMI 22.3±0.4 kg/m^2^) were included in the study. Obese subjects were treated with a very low-calorie diet (approximately 800 kcal/d) for 12 weeks. Body composition was assessed by impedance analysis, insulin sensitivity was estimated by HOMA-IR and the leptin-to-adiponectin ratio and wnt5a and sFRP5 serum concentrations were measured by ELISA. sFRP5 expression in human adipose tissue biopsies was further determined on protein level by immunohistology.

**Principal Findings:**

Pro-inflammatory wnt5a was not measurable in any serum sample of lean control subjects. In patients with obesity, however, wnt5a became significantly detectable consistent with low grade inflammation in such subjects. Caloric restriction resulted in a weight loss from 131.9±4.0 to 112.3±3.2 kg in the obese patients group. This was accompanied by a significant decrease of HOMA-IR and leptin-to-adiponectin ratio, indicating improved insulin sensitivity. Interestingly, these metabolic improvements were associated with a significant increase in serum concentrations of the anti-inflammatory factor and wnt5a-inhibitor sFRP5.

**Conclusions/Significance:**

Obesity is associated with elevated serum levels of pro-inflammatory wnt5a in humans. Furthermore, caloric restriction beneficially affects serum concentrations of anti-inflammatory sFRP5 in such subjects. These findings suggest a novel regulatory system in low grade inflammation in obesity, which can be influenced by nutritional therapy.

## Introduction

During the last decades the mean body mass index (BMI) is increasing continuously in most countries within the western world making obesity one of the most important health problems. Especially in patients with co-morbidities, such as hypertension and type 2 diabetes, low grade inflammation has been observed in the absence of infections or classical immunological diseases [1]. Several recent reports in cell lines [2], animal models [3] and humans [4], [5] suggest that these inflammatory reactions are not only associated with obesity but are causally involved in the pathogenesis of obesity and its co-morbidities.

Macrophages in adipose tissue are known to secrete pro-inflammatory cytokines like tumour necrosis factor (TNF)-α which have been shown to alter the function of mature adipocytes and the differentiation of preadipocytes in animal models and cell culture systems [6], [7]. It is thought that due to reduced adipogenesis the storage capacity of inflamed adipose tissue is reduced resulting in ectopic lipid accumulation in liver and skeletal muscle leading to insulin resistance of these metabolically important tissues and eventually type 2 diabetes [8], [9]. However, in human subjects treatment with the TNF-α antibody adalimumab [10] or the soluble TNF-α receptor etanercept [11] did not significantly improve insulin sensitivity suggesting that in humans macrophages might affect adipose tissue by different bioactive molecules.

In a recent report from our group we identified the secreted glycopeptide *wingless-type MMTV integration site family member* (wnt)-5a as a potent inhibitor of adipogenesis in human mesenchymal stem cells [12]. Furthermore, we found that adipose tissue macrophages of obese and type 2 diabetic human subjects express wnt5a *in vivo* and that wnt5a secreted by macrophages inhibits differentiation of preadipocytes [13]. These recent findings suggest that wnt5a might act as an important pro-inflammatory molecule in low grade inflammation of adipose tissue in obese humans.

The *soluble frizzled related protein* (sFRP)-5 is a known inhibitor of wnt5a signalling [14]. Recently it has been shown in mice that healthy adipocytes are able to secrete sFRP5 to protect themselves from wnt5a. In diet induced obesity, sFRP5 expression in adipose tissue was found to be up-regulated in animal models [14], [15]. However, this effect might only be transient, since Ouchi et al. found sFRP5 levels to fall below control levels under conditions of severe metabolic dysfunction in relation to obesity [14]. Furthermore, it has been shown in animal models that sFRP5 expression in adipose tissue is altered by nutritional intervention [15].

Until now data on wnt5a and sFRP5 in human subjects with obesity are limited. Since both bioactive molecules can be detected in blood samples by ELISA we aimed to address two major questions in the present clinical study: (1) are wnt5a serum concentrations increased in blood samples of human subjects with severe obesity compared to lean controls and (2) does caloric restriction either reduce wnt5a and/or improve sFRP5 serum levels which would suggest a beneficial effect on low grade inflammation in obesity.

## Materials and Methods

### Study populations

#### Adipose tissue biopsy study population

The study was approved by the ethics committee of the University of Cologne and written informed consent was obtained from all participants. Subcutaneous adipose tissue biopsies were taken during elective surgical procedures from n = 5 lean subjects (age: 52,4±4,74, 60% males, mean BMI 24,0 kg/m^2^). Exclusion criteria were: acute or chronic infectious or immunological disease, cancer, elevation of liver function tests >3-fold of normal range, serum-creatinin levels >1.5 mg/dl, hyper- and hypocortisolism, hyper- or hypothyroidism and lipodystrophy.

#### Nutritional intervention study population

The study was approved by the ethics committee of the University of Cologne. Written informed consent was obtained from each subject before inclusion into the study. A total number of n = 12 metabolically healthy control subjects were included. The obesity treatment group consisted of n = 23 human subjects. As shown in table 1, the groups were equally distributed regarding age and gender while body weight and body mass index (BMI) were significantly different. Inclusion criteria were: age between 20 and 65 years and caucasian descent. Exclusion criteria were: acute or chronic infections, presence of cancer, elevations of liver function tests >3-fold of normal range, serum-creatinin levels >1.5 mg/dl, pregnancy, untreated hypo- and hyperthyroidism, hypo- and hypercortisolism and growth hormone deficiency. In the obesity group 2 patients were smokers, 2 patients were suffering from type 2 diabetes which was sufficiently controlled only by metformin therapy (metformin was discontinued during VLCD), 7 patients had hypertension, 4 patients had hyperlipidemia, 1 subject had sleep apnoea syndrome, 1 patient had gastroesophageal reflux disease, 2 patients had recurrent phlebothrombosis in the past, 7 patients had hypothyreoidism which was controlled by thyroxine treatment, 2 patients had allergic asthma, 1 patient had history of acute hepatitis A. None of the control subjects were taking pharmacotherapy for any kind of diseases. In terms of the obesity group, 7 patients were taking thyroxine, 2 patient were taking ACE-inhibtors, 5 patients were taking AT1-blockers, 1 patient was taking a renin inhibitor, 4 patients were on diuretics, 2 patients were taking beta-blockers, 1 patient was taking a Ca^2+^ channel blocker, 2 patients were on phenprocoumon, 2 patients were taking acetylsalicylic acid, 2 patients were taking inhalative beta-sympathomimetics, 2 patients were taking inhalative steroids, 2 patients were on metformin therapy which was discontinued during VLCD, 2 patients were taking anti-depressant agents, 1 patient was taking an antacid and 2 female patients were taking hormonal contraception.

**Table 1:** Baseline characteristics of the control and the obesity study group.

	lean	obese	p-value
gender (% female)	66.7	65.2	p = 0.934
age (years)	37.8±3.2	42.8±2.6	p = 0.242
weight (kg)	66.5±2.8	131.9±4.0	p<0.001
BMI (kg/m^2^)	22.3±0.4	44.1±1.1	p<0.001
fasting insulin (µU/ml)	10.0±2.2	17.2±1.9	p<0.01
fasting glucose (mg/dl)	81.9±4.3	92.7±3.8	p<0.05
fasting triglycerides (mg/dl)	109.4±11.7	146.9±11.8	p<0.05
C reactive protein (mg/l)	2.1±1.4	7.2±1.0	p<0.01
HOMA-IR index	1.9±0.3	3.9±0.6	p<0.01

### Immunohistology

Human adipose tissue biopsies were fixed in 4% paraformaldehyd/phosphate-buffered saline solution for 24 h at 4°C. Samples were dehydrated by ascending ethanol series and embedded in paraffin. Paraffin sections of 4 µm thickness were assembled and dried on tissue slides. Deparaffinisation was achieved by xylene and 100% ethanol following rehydration by descending ethanol series. During hydration, a 5 minute blocking for endogenous peroxidase was done with 0.3% H_2_O_2_ in 95% ethanol. Rehydrated samples were washed with phosphate-buffered saline containing 0.2% TWEEN® 20. Prior to over-night incubation with sFRP5 antibody (HPA019840; Sigma Aldrich) at 4°C, an antigen retrieval was performed using sodium citrate buffer (pH6). Subsequently, samples were washed 2×5 min in phosphate-buffered saline containing 0.2% TWEEN® 20 and incubated with a second horseradish peroxidase-conjugated antibody (DAKO EnVision+ System-HRP Labelled Polymer Anti-Rabbit; DAKO) for 30 min at room temperature (RT). Positive cells were detected by applying DAKO AEC Substrate Chromogen (DAKO) for approximately 15 min at RT. Nuclear staining was done using haemotoxylin (Mayer's Haemalaun-Solution; AppliChem GmbH). Tissue slides were then coated with gelatine (Kaiser's glycerol gelatine; Merck KGaA) and analysed by microscopy.

### Nutritional intervention

Formula diet was obtained from Nestle Nutrition (Optifast©). Total caloric intake was approximately 800 kcal/d divided into 4–5 meals/d. Formula diet was embedded into a commercial multimodal obesity program which also included nutritional education, behavioural advice and exercise courses (Optifast-52© program, www.optifast.de).

### Body composition analysis

Body composition was measured by impedance analysis using the Tanita Body composition analyser BC-418 MA.

### Biochemical analysis

Blood samples were taken after an overnight fast at 0, 4 and 12 weeks. The following ELISA for adipokine and wnt-signalling molecules were used: Leptin: IBL international, order number RE53151 (analytical-sensitivity: 1.0 ng/ml)), Adiponectin: IBL international, order number: BV51001 (analytical-sensitivity: 0.2 µg/ml), wnt5a: Uscn Life Science Inc., order number E83549Hu, (analytical-sensitivity: 0.051 ng/ml), sFRP5: (analytical-sensitivity:0.64 ng/ml) Uscn Life Science Inc., order number E92842Hu (analytical-sensitivity:0.64 ng/ml). The ELISAs were performed according to the instructions of the manufacturer. According to the manufacturer, both the wnt5a ELISA and the sFRP5 ELISA had excellent specificity for detection of human wnt5A and sFRP5 and no significant cross-reactivity or interference with analogues was observed. The specificity of the antibodies was confirmed by western blot experiments. For the wnt5a antibody, two cell line lysates were tested. The positive one were Hela cells and the negative one were HEK293. For the sFRP5 antibody, tissue homogenates from human pancreas and liver tissue were tested separately (personal communications with the supplying company).

### Statistical analysis

Statistical analysis was performed using student's t-test. p<0.05 was considered to be significant. Data are given as means ± SEM. * p<0.05, ** p<0.01, *** p<0.001.

## Results

### sFRP-5 is expressed in mature adipocytes in human subjects *in vivo*

**Figure 1:**
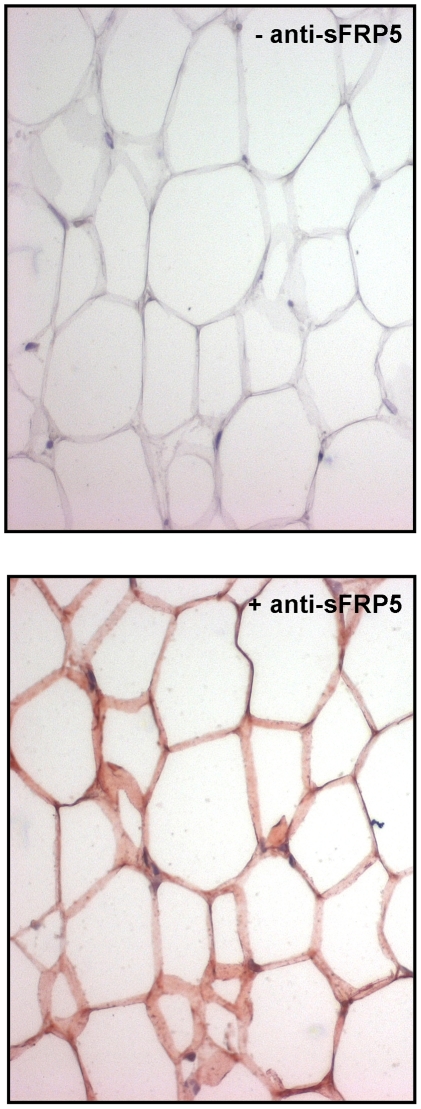
sFRP5 expression in human adipose tissue. Immunohistochemistry for sFRP5 and hematoxylin staining of human subcutaneous adipose tissue biopsies. One representative example of n = 5 lean control subjects. Left panel: haematoxylin staining, right panel: immunohistochemistry for sFRP5. The reddish colour represents a positive result for expression.

In a recent report we have shown that wnt5a is expressed in adipose tissue macrophages in humans [13]. However, data on sFRP5 expression in humans to the best of our knowledge have been shown only on RNA level in whole adipose tissue biopsies, which contain all types of different cells present in adipose tissue, such as mature adipocytes, preadipocytes, macrophages, endothelial cells, lymphocytes etc. In order to examine if mature adipocytes express sFRP5 in adipose tissue in humans, we first performed immunohistochemistry for sFRP5 in subcutaneous fat biopsies of lean human control subjects. As shown in fig. 1, these experiments clearly revealed that sFRP5 is expressed in the cytoplasm of mature adipocytes.

### wnt5a serum concentrations are increased in obese human subjects

**Figure 2:**
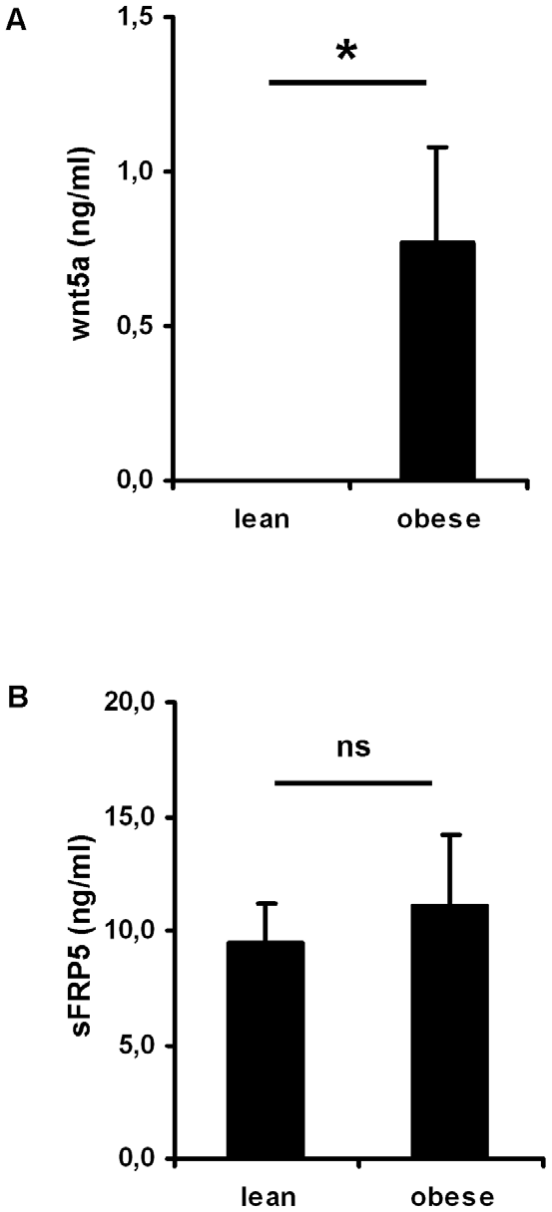
wnt5a and sFRP5 serum concentrations in lean control subjects and patients with obesity. Serum samples were taken after an overnight fast and wnt5a and sFRP5 serum concentrations were measured by ELISA. Shown are means±SEM for n = 12 lean control subjects and n = 23 patients with obesity. In order to test for statistical significance, student's t-test was used, ns = not significant.

We next measured wnt5a and sFRP5 protein concentrations in serum samples of n = 12 lean control subjects and n = 23 subjects with obesity. As shown in table 1, both groups were equally distributed in terms of age and gender while body weight and BMI were almost doubled in the obesity group compared to the lean control group. Obese subjects exhibited a HOMA-IR index above 2 indicating insulin resistance. Furthermore, C reactive protein levels (CRP) in the obese group were above the normal range (<5 mg/l) and were significant higher compared to lean controls indicating low grade inflammatory activity (tab. 1). wnt5a was not detectable in any serum samples of the control group. In obese human subjects, however, wnt5a was significantly measurable with serum concentrations of 0,77±0,31 ng/ml (fig. 2). We next measured sFRP5 protein concentrations in the same serum samples. In contrast to what was found for wnt5a, sFRP5 was detectable in lean (9,47±1,75 ng/ml) and obese subjects (11,04±3,15 ng/ml) with no significant difference between the groups (fig. 2).

### Caloric restriction increases sFRP5 serum concentrations in obese human subjects

**Figure 3:**
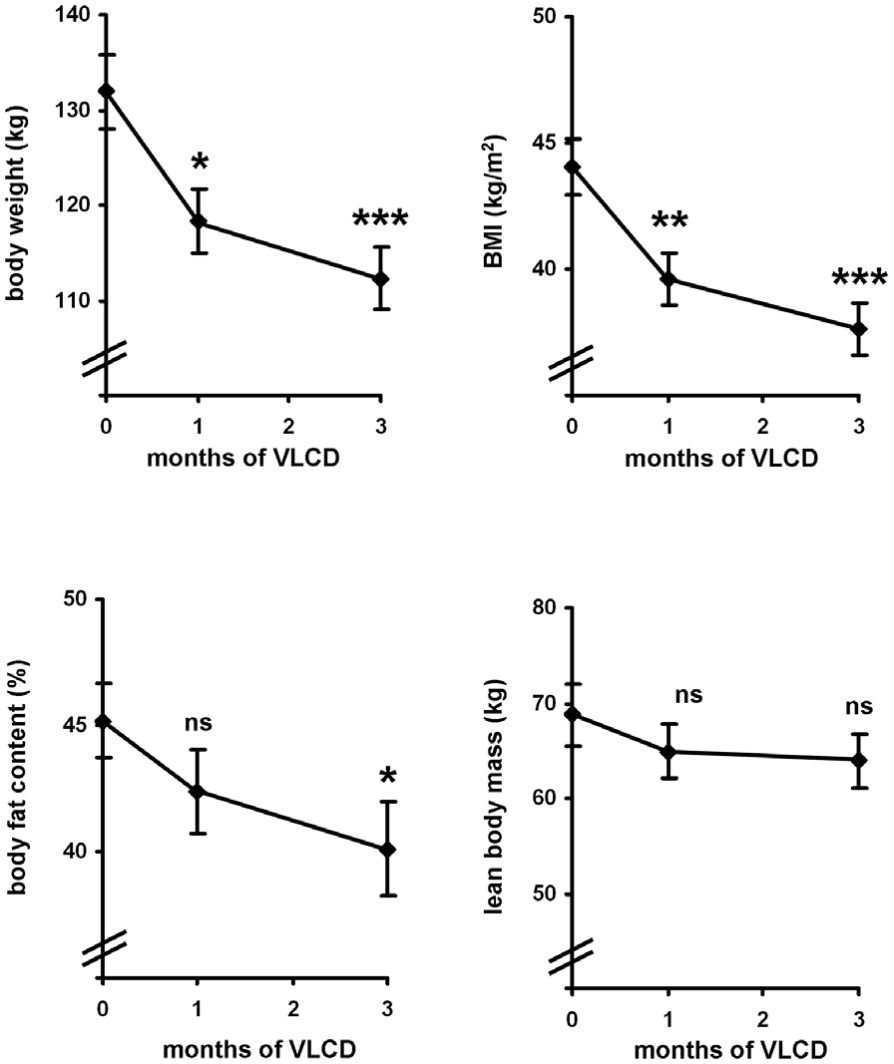
Anthropometric measurements before, during (1 month) and after 3 months of caloric restriction: Body composition was estimated by impedance analysis. Shown are means±SEM of n = 23 obese human subjects during a VLCD. Statistical significance was tested by student's t-test, ns = not significant.

Having shown that both, wnt5a and sFRP5 are detectable in serum samples of obese human subjects we next examined, if these bioactive peptides can be influenced by caloric restriction. Therefore we treated the 23 patients of the obesity group with a very low calorie formula diet (VLCD) for 12 weeks (fig. 3). This treatment resulted in a significant loss of body weight (131.9±4.0 to 112.3±3.2 kg, p<0.001) and BMI (44.1±1.1 to 37.6±1.0 kg/m^2^, p<0.001). The weight loss was mainly due to loss of fat mass which was significantly reduced (45.2±1.5% to 40.1±1.6%, p<0.05) while lean body mass was not significantly altered (68.7±3.4 to 64.0±2.8 kg, p = 0.282) (fig. 3). However, it has to be mentioned that even after the caloric restriction obese subjects had still a BMI>35 kg/m^2^ and an average body fat content of 40% (fig. 3).

**Figure 4:**
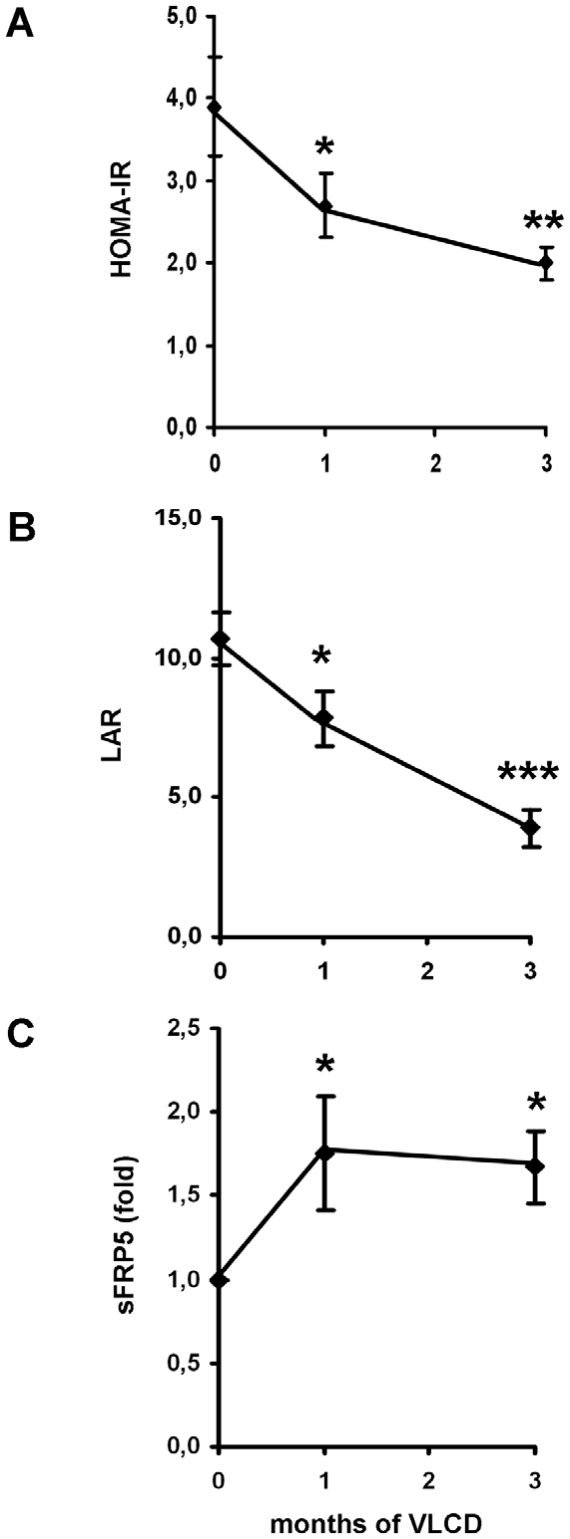
(A+B) Insulin resistance before, during (1 month) and after 3 month of caloric restriction: In the present study insulin resistance was measured by the HOMA-IR index (A) and the leptin-to-adiponectin ration (LAR) (B) since the later parameter has been shown to be closely correlated to measures of insulin resistance by hyperinsulinemic euglycemic clamp experiments [**16**]. Data are given as means±SEM of n = 23 obese human subjects during a VLCD. Significance was tested by student's t-test. (**C**) *sFRP serum concentrations before, during (1 month) and after 3 months of caloric restriction*: sFRP5 serum concentrations were determined before (0 month), during (1 month) and after (3 months) of a VLCD. In this figure the levels are shown as fold increase compared to 0 month. The raw data are given in table 2. Shown are means±SEM of n = 23 obese human subjects during a VLCD. t-test was used to test for statistical significance.

In order to estimate insulin resistance during caloric restriction we measured HOMA-IR index which exhibited a significant reduction throughout the treatment period (fig. 4A). We also measured leptin and adiponectin serum concentrations (tab. 2) and calculated the leptin-to-adiponectin ratio (LAR). This marker was recently shown to be a suitable measure of insulin sensitivity with a close correlation to hyperinsulinemic euglycemic clamp data [16]. As shown in figure 4B, LAR significantly decreased from 10.7±1.0 to 3.9±0.7 (p<0.001) indicating improvement of insulin sensitivity due to weight loss by caloric restriction. CRP levels were not significantly altered during the VLCD (before: 7.2±1.0 mg/l, after 6,7±1.8 mg/l (p = 0.424)). Wnt5a and sFRP5 serum samples were measured before, during (1 month) and after 3 month of VLCD. As shown in table 2, weight loss and improved insulin sensitivity did not result in a significant change in wnt5a serum concentrations. The wnt5a inhibitor sFRP5, however, was significantly increased by approximately 70% already after 4 weeks of VLCD. This increase was sustained to the same extent till the end of the study period (fig. 4C). Finally we compared wnt5a and sFRP5 values between subjects with weight loss >15% (n = 10) and subjects with weight loss <15% (n = 13) and found no significant difference (p = 0.245 for wnt5a and p = 0.342 for sFRP5).

**Table 2:** Adipokine, wnt5a and sFRP5 serum concentrations before, during (1 month) and after 3 months of caloric restriction.

	before	during	after
adiponectin µg/ml)	6.1±0.6	6.0±0.4	7.1±0.6
leptin (ng/ml)	61.7±6.1	46.4±6.3	27.0±5.3
wnt5a (ng/ml)	0.8±0.3	0.6±0.2	0.8±0.4
sFRP5 (ng/ml)	11.0±3.2	17.5±4.9	14.8±4.4

## Discussion

In the year 2000 the first report on wnt signalling being important in the molecular regulation of adipogenesis has been published [17]. Wnt's are secreted glycopeptides that can act in an autocrine or paracrine manner. Upon binding to specific receptors wnt molecules can activate different intracellular pathways which are referred to as “canonical” and “non-canonical” signalling. Activation of both pathways results mainly in changes of gene expression in target cells (for review see [18]). Wnt signalling can also be specifically inhibited by several biochemical mechanisms. S*oluble frizzeld-related proteins* (sFRP) can bind to wnt molecules and thereby sequester them from their cell surface receptors [19]. The soluble factor *Dickkopf* (DKK)-1 also acts extracellularly by binding to the LRP co-receptor resulting in a specific inhibition of the canonical pathway [20]. Besides that, wnt signalling can also be inhibited within the cell either in the cytoplasm [21] or in the nucleus [22].

Several members of the wnt family have been shown to inhibit differentiation of mesenchymal precursor cells into mature adipocytes in cell culture [23], animal models [24] and humans [25]. For example, wnt5a inhibits adipogenesis of human mesenchymal stem cells *ex vivo* by activating the JNK dependent non-canonical pathway [12]. In agreement it has been shown in an animal model, that alterations in the locus of the wnt5a, the JNK1 and the PKC-delta gene are associated with obesity, suggesting that wnt5a acts as an important anti-adipogenic factor not only in cell culture systems but also in a whole body organism [26].

Of the many wnt molecules and inhibitors shown to function in the regulation of adipogenesis, wnt5a and its inhibitor sFRP5 are particularly important since they both have recently been implicated in low grade inflammation of adipose tissue. Our own group found wnt5a to be expressed in adipose tissue macrophages in human subjects with obesity and type 2 diabetes *in vivo* [13]. Moreover, wnt5a secreted from these inflammatory cells was shown to inhibit differentiation of preadipocytes into mature fat cells, suggesting wnt5a to act as a pro-inflammatory factor inhibiting hyperplasia of adipose tissue [13]. Interestingly, in a recent report of an independent group the wnt5a inhibitor sFRP5 was identified as an anti-inflammatory factor secreted by adipocytes of lean animals [14]. In the same study two independent mouse models of obesity exhibited reduced sFRP5 expressions and an increased wnt5a/sFRP5 ratio in adipose tissue [14] suggesting these two bioactive molecules to be involved in the development of obesity and type 2 diabetes. Together, these findings fit nicely in the concept of the “overflow-hypothesis”, in which a reduced storage capacity of adipose tissue due to limited expansion leads to ectopic lipid accumulation in liver and skeletal muscle which is crucial in inducing insulin resistance [8], [9]. Since in humans, sFRP5 has been shown to be expressed on RNA level in whole adipose tissue biopsies but not in individual mature adipocytes we performed immunohistology of subcutaneous fat biopsies in the present study in order to examine in which type of cells sFRP5 is expressed. These experiments revealed that sFRP5 is indeed expressed in the cytoplasm of mature adipocytes in humans *in vivo* (fig. 1).

As mentioned above, wnt signalling normally occurs in an autocrine or paracrine manner in different tissues and no major endocrine function has been reported so far. However, in sepsis wnt5a was shown to be detectable in serum samples of affected subjects, suggesting that under special circumstances wnt5a might be released by inflamed tissues into the circulation [27]. Inflammation associated with obesity is by far of a lower grade compared to the condition of sepsis, however, especially in severe obese individuals the amount of adipose tissue as a potential source of wnt5a is extremely increased. Therefore we aimed to investigate if wnt5a becomes detectable in serum samples in such individuals. As a control group in our study we included 12 normal weight individuals of same age and gender distribution. As expected, in none of these lean control individuals wnt5a was detectable, confirming that it preferentially acts as an autocrine and/or paracrine factor but not as an endocrine signalling molecule. In patients with severe obesity, however, wnt5a was detectable in serum samples in the absence of any acute or chronic classical inflammatory or immune disease, which might suggest that wnt5a can be released from inflamed adipose tissue into systemic circulation. In contrast to wnt5a, sFRP5 serum concentrations were detectable in the lean control group as well as in the group of severe obese subjects. The serum concentrations measured were not significant different between both groups. However, it has to be taken into account that the mean body weight of the obese patients group in the present study was almost doubled compared to the lean control group with most of the difference account to increase in adipose tissue mass (fig. 3). Taking this into consideration, it can be speculated that the amount of sFRP5 released per fat cell is reduced in obese human individuals.

In the present study we also examined the effect of caloric restriction on wnt5a and sFRP5 serum concentrations in obese human subjects. Within the three months of the study period, wnt5a levels were not significantly reduced by caloric restriction despite a significant weight loss and improved insulin sensitivity (tab. 2). In contrast to pro-inflammatory wnt5a, serum concentrations of anti-inflammatory sFRP5 exhibited a fast response to caloric restriction. Already after 4 weeks sFRP5 levels were significantly increased (fig. 4C). Together, these findings suggest that sFRP5 is more sensitive to nutritional therapy compared to wnt5a which might have clinical implications, e. g. the use of these bioactive molecules as biomarkers for anti-inflammatory effects of dietary interventions.

In summary, the results of the present study together with the recent reports in humans and mice on wnt signalling in adipose tissue suggest an interplay of pro-inflammatory wnt5a released from macrophages and anti-inflammatory sFRP5 secreted by adipocytes in obese human subjects. These findings might be of clinical relevance in the future since sFRP5 can be beneficially influenced by nutritional therapies, as shown in this interventional study.
